# Correction

**DOI:** 10.1080/19491034.2024.2443274

**Published:** 2025-01-21

Article title: Locus-specific differential expression of human satellite sequences in the nuclei of cancer cells and heat-shocked cells

Authors: C. Rabeler., N. Paterna., R. Potluri., L. R. D’Alessandro., A. Bhatia., S. Y. Chen., J. Lee., B. Abeje., B. Lipchin., B. R. Carone., D. M. Carone

Journal: ***Nucleus***

Bibliometrics: Volume 15, Number 01, pages 01 - 19

DOI: 10.1080/19491034.2024.2431239

This article was previously published with a mistake in units. This has now been added and republished in the original article.

- Mm to mM
- uM to μm

